# Mismatch Between Imaging and Histopathology Diagnosis in High and Low-Grade Gliomas: A Case Report

**DOI:** 10.31729/jnma.v63i292.9261

**Published:** 2025-12-31

**Authors:** Pukar Adhikari, Jabir Ahamad Miya, Shishir Bhandari, Ganesh Adhikari, Ajit Shrestha, Nikesh Tiwari

**Affiliations:** 1 Chitwan Medical College Teaching Hospital, Bharatpur, Chitwan, Nepal

**Keywords:** *glioma*, *IDH mutation*, *oligodendroglioma*, *seizure*

## Abstract

Oligodendrogliomas aie diffusely infiltrating gliomas classified as WHO grade 2 or 3 tumors. While typically presenting with chronic seizures, they may rarely demonstrate imaging features suggestive of high-grade malignancy. A 40-year-old lady with untreated seizures for one year presented with acute loss of consciousness and recurrent generalized tonic-clonic seizures. Neuroimaging (CT/MRI) revealed a left temporoparietal lesion with cystic changes and hemorrhage, suggesting high-grade glioma. Histopathological analysis following craniotomy unexpectedly demonstrated WHO Grade 2 oligodendroglioma. This case highlights significant radiologic-histopathologic discordance in glioma diagnosis, emphasizing that radiologic features of high-grade malignancy may occur in low-grade oligodendrogliomas. Tissue diagnosis with molecular profiling remains essential for accurate classification and management.

## INTRODUCTION

Gliomas, the most common primary CNS tumors, arise from glial cells and are classified by the WHO into low-grade glioma (grade 1-2) and high-grade glioma (grade 3-4) based on histopathology and molecular markers including IDH mutation and 1p/19q codeletion.^1-4^ While low grade glioma, often present with seizures in young adults, imaging findings can sometimes conflict with histopathology, delaying accurate diagnosis and treatment, a critical challenge in settings where advanced molecular Judies are not feasible.^5^ We highlight such a discrepancy in this case, underscoring the need for integrated diagnostic approaches to optimize patient outcomes.

## CASE REPORT

A 40-year-old female with a one-year history of untreated seizure disorder presented to the emergency department with acute onset loss of consciousness and multiple episodes of generalized tonic-clonic seizures within a 24-hour period. The ictal events were characterized by limb stiffness, frothing from the mouth, leftward mouth deviation, urinary incontinence, and teeth clenching. Postictal confusion was present for a few minutes. She also had fever with a maximum temperature recorded was 102.3°F at the time of presentation.

On examination, the patient had a GCS of 12/15 (E4V3M5), pinpoint pupils bilaterally, and was agitated and disoriented.

**Figure 1:**
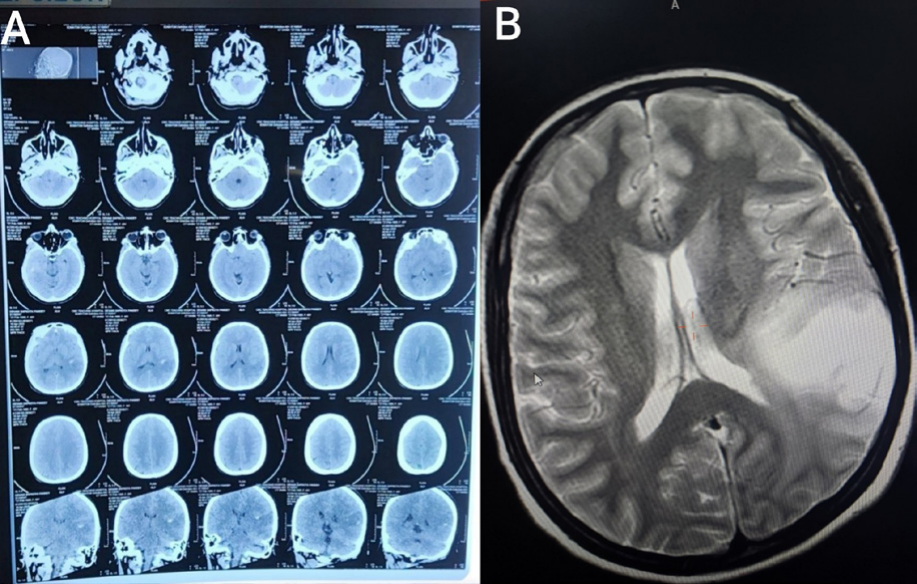
Pre-operative imaging studies. 1(A) Non contrast CT head. 1(B) MRI brain.

She was right-handed. The motor power was symmetrically reduced (4/5 in all limbs) with bilateral upgoing plantar reflexes. There were no signs of meningeal irritation. On investigation, the complete blood count, renal function test, blood glucose level, ECG, serum electrolytes were within normal limit. Initial imaging with CT head, as shown in figure 1 (A), revealed poorly outlined hypodensity involving both grey and white matter on the left temporo-parietal lobe with small focus of hemorrhage and few cystic areas, no calcifications seen and effaced adjacent sulci. These findings most likely suggested neoplasm.

The MRI Brian, as shown in figure 1 (B), showed ill-defined mass in the cortical and sub-cortical white matter of left temporal and parietal lobe. Significant perilesional edema was noted. Mild mass effect seen in the form of effacement of adjacent cortical sulci in para-falcine area. She underwent left temporoparietal craniotomy with tumor evacuation. The operative findings revealed that tumor was soft, greyish, moderately vascular, and non-encapsulated. Areas of haemorrhage were noted within the tumor region. After successful removal, the tumor was sent for histopoathological analysis in the Department of Pathology, Chitwan Medical College. Post-operatively, the patient required mechanical ventilation and supportive ICU care, followed by a stable recovery and discharge.

**Figure 2:**
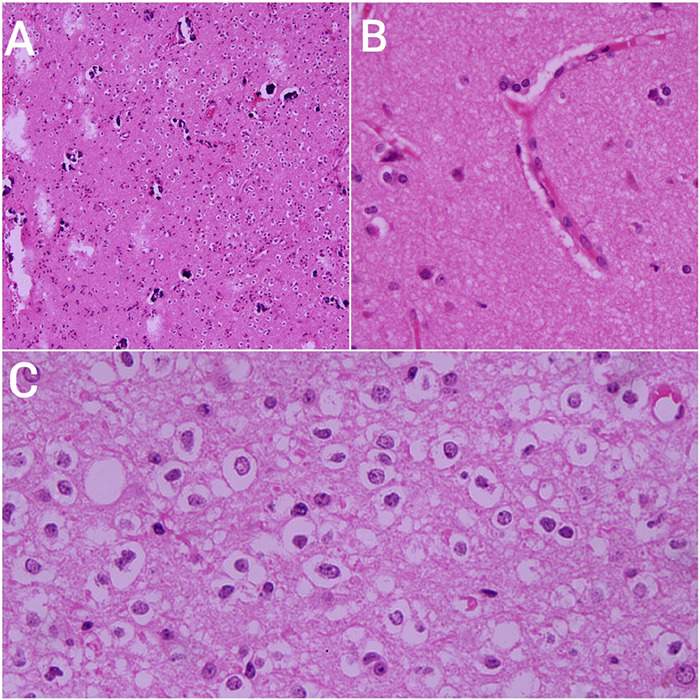
Histopathological findings A: Uniform round nuclei with perinuclear halos showing the classic “fried egg” appearance in a delicate fibrillary background. B: Prominent branching capillaries creating a “chicken-wire” vascular pattern, typical of oligodendroglioma histology. C: Monomorphic cells with clear cytoplasm and round nuclei; mild nuclear atypia without mitosis or necrosis, consistent with grade 2 tumor.

**Figure 3:**
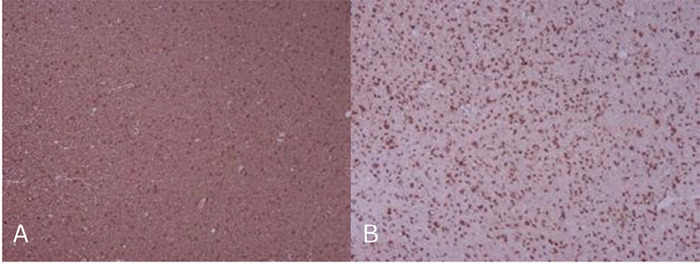
Immunohistochemistry A: GFAP B: IDH-1 Representative image showing strong immunoreactivity for IDH-1 (3+), GFAP (4+), and Olig-2 (4+) in lesional cells.

The histopathology results showed findings as shown in figure 2; uniform round nuclei with perinuclear halos showing the classic “fried egg” appearance of oligodendroglioma with prominent branching capillaries creating a “chicken-wire” vascular pattern. These features suggested WHO Grade 2 oligodendroglioma. Immunohistochemistry was advised by the pathology department and hence an sample was sent to the outside centre. Immunohistochemistry demonstrated findings as shown in figure 3; strong immunoreactivity for GFAP (4+), Olig-2 (4+), and IDH-1 (3+) in the lesional cells. The Ki-67 proliferative index was relatively low, ranging between 8-10%, indicating a low to moderate proliferative activity. ATRX expression was retained, and patchy loss of H3K27me3 was observed. The overall morphology, in conjunction with this immunoprofile, supports the diagnosis of oligodendroglioma. The patient was discharged in a stable condition and advised for follow-up in neurosurgery OPD with recommendation for further molecular studies including 1p/19q codeletion and IDH sequencing for definitive classification.

On follow up, the patient was asymptomatic and compliant to oral antiseizure drugs. Further molecular studies were declined by the patient and her family due to financial constraints.

## DISCUSSION

Oligodendrogliomas are rare primary brain tumors that generally present with chronic symptoms such as focal seizures or headaches.^2^ However, our patient’s acute presentation with prolonged unconsciousness, fever and recurrent generalized seizures posed a diagnostic challenge.^5^ Initial imaging suggested a high-grade glioma due to the heterogeneous nature of the lesion and associated hemorrhagic and cystic components. However histopathology following craniotomy revealed a WHO Grade 2 oligodendroglioma with favorable molecular features, including IDH-1 positivity, retained ATRX, and a low proliferative index.^3,6^ This significant mismatch between radiological impression and histological diagnosis underscores the complexity of glioma grading and its implications for treatment.

This case highlights several key learning points. First, glioma grading is crucial, as it determines prognosis and guides therapeutic decisions. While high-grade gliomas often require urgent and aggressive treatment, ‘low-grade gliomas’ may be managed with more conservative or staged approaches.^7^ In our case, the radiologic mimicry of a high-grade lesion led to an expedited surgical decision^8^ left temporoparietal craniotomy with tumor evacuation; which ultimately proved therapeutic, but could have been preceded by other diagnostic options.^1^ Advanced MRI modalities such as perfusion-weighted imaging, MR spectroscopy, or functional MRI where available might have offered additional clues to the tumor’s true nature.

Second, while surgical intervention was justified due to mass effect and ongoing seizures, earlier consideration of MRI-guided stereotactic biopsy could have helped establish diagnosis less invasively.^9^ Studies show that stereotactic biopsy carries a diagnostic accuracy of approximately 70-80% and may reduce unnecessary surgical morbidity in selected patients.^10^ First-pass gadopentetate dimeglumine enhanced echo-planar perfusion MRI may be valuable as a complementary technique for the preoperative assessment of high- and ‘low-grade gliomas’ in selected cases. Unfortunately, this was not feasible in our setting at the time, and the decision for surgery was made based on the acute clinical deterioration and imaging findings. Also, extended surgery was shown to significantly prolong overall survival in glioma patients which justifies our decision for expedited surgical removal of the tumor.^11^

Another important aspect is the lack of early seizure control. The patient had a year-long history of untreated seizures and presented with status epilepticus.^12^ Prompt initiation of anti-seizure medications such as levetiracetam, which has shown favorable outcomes in glioma-associated epilepsy, might have stabilized the patient prior to surgery.^13^ This case emphasizes the need for early neurological assessment and consistent follow-up in patients with chronic seizures, which can prevent delay in the diagnosis of underlying pathology.

Referral to ahighercenter for advancedneurodiagnostic or interventional procedures could also have been considered earlier. In resource-limited settings, this often remains a challenge due to infrastructure, affordability, and geographical barriers. Nonetheless, multidisciplinary evaluation and timely referral can significantly impact patient outcomes in complex neuro-oncology cases.^14^ We also acknowledge the growing interest in Ayurvedic adjuncts for supportive care in brain tumors. Though not curative, herbal interventions such Káñcanára Guggulu, Guducl Sattva, Septillin tablets, and Asvagandhand have been studied for their neuroprotective and antioxidant properties. While evidence remains preliminary, integration of such therapies under professional guidance may contribute to symptom relief and quality of life in selected patients.

## CONCLUSION

Oligodendrogliomas can present with features mimicking high-grade gliomas, including hemorrhage and mass effect. Radiology alone may not reliably indicate tumor grade, necessitating early neuro-interventional or surgical biopsy and molecular analysis for accurate classification. Early evaluation of chronic seizure disorders is critical for timely diagnosis and management.

## References

[1] Campos LG, de Oliveira FH, Antunes ÁCM, Duarte JÁ (2024). Evaluation of Glial Tumors: Correlation Between Magnetic Resonance Imaging and Histopathological Analysis.. Radiol Bras..

[2] Forst DA, Nahed BV, Loeffler JS, Batchelor TT (2014). Low-Grade Gliomas.. Oncologist..

[3] Skouras P, Giakoumettis G, Argyros C, Vavoulis G, Verigos EK, Giakoumettis D (2025). Oligodendroglioma of the Hippocampus: A Case Report and Systematic Review on Therapeutic Approaches of Oligodendroglioma After WHO 2021 Classification.. Pharmaceuticals (Basel)..

[4] Carstam L, Rydén I, Jakola AS (2022). Seizures in Patients with IDH-Mutated Lower Grade Gliomas.. J Neurooncol..

[5] Aguilar-Hidalgo KM, Alvarez-Castro JA, Santelián-Hernández JO, Calderón-Garcidueñas AL, Romero-Luna G, Monjarás-Romo G (2022). A Patient with Epilepsy, Ganglioglioma, and Oligodendroglioma with Anaplastic Foci in the Same Left Frontoparietal Lesion: A Case Report.. Cureus..

[6] Kros JM (1995). Oligodendrogliomas: Clinicopathological Correlations.. J Neurooncol..

[7] Aboud O, Shah R, Vera E, Burton E, Theeler B, Wu J (2022). Challenges of Imaging Interpretation to Predict Oligodendroglioma Grade: A Report from the Neuro-Oncology Branch.. CNS Oncol..

[8] Duffau H (2013). A New Philosophy in Surgery for Diffuse Low-Grade Glioma (DLGG): Oncological and Functional Outcomes.. Neurochirurgie..

[9] McGirt MJ, Villavicencio AT, Bulsara KR, Friedman AH (2003). MRI-Guided Stereotactic Biopsy in the Diagnosis of Glioma: Comparison of Biopsy and Surgical Resection Specimen.. Surg Neurol..

[10] Hakyemez B, Erdogan C, Ercan I, Ergin N, Uysal S, Atahan S (2005). High-Grade and Low-Grade Gliomas: Differentiation by Using Perfusion MR Imaging.. Clin Radiol..

[11] Sharma S, Jain R (2022). Dilemma in Low-Grade Glioma Surgery: Review of Literature and When to Operate.. Int J Neurosurg..

[12] Breemen MSM, Rijsman RM, Taphoorn MJB, Walchenbach R, Zwinkels H, Vecht CJ (2009). Efficacy of Anti-Epileptic Drugs in Patients with Gliomas and Seizures.. J Neurol..

[13] De Bruin ME, Van Der Meer PB, Dirven L, Taphoorn MJB, Koekkoek JAF (2021). Efficacy of Antiepileptic Drugs in Glioma Patients with Epilepsy: A Systematic Review.. Neurooncol Pract..

[14] Raman S, Ashwini BN, Sivabalaji K (2021). A Case Report on Grade II Oligodendroglioma by Ayurvedic Intervention.. J Nat Remed..

